# Verticillium Wilt in Oilseed Rape—the Microbiome is Crucial for Disease Outbreaks as Well as for Efficient Suppression

**DOI:** 10.3390/plants9070866

**Published:** 2020-07-08

**Authors:** Daria Rybakova, Mariann Wikström, Fia Birch-Jensen, Joeke Postma, Ralf Udo Ehlers, Maria Schmuck, René Kollmann, Jürgen Köhl, Gabriele Berg

**Affiliations:** 1 Graz University of Technology, Environmental Biotechnology, Petersgasse 12, 8010 Graz, Austria; daria.rybakova@tugraz.at (D.R.); laborschmuck@aon.at (M.S.); 2 Agro Plantarum AB, Kärrarpsvägen 410, S-265 90 Åstorp, Sweden; mariann.wikstrom@agroplantarum.se (M.W.); fia.birch-jensen@agroplantarum.se (F.B.-J.); 3 Wageningen University & Research, 6708 PB Wageningen, Netherlands; joeke.postma@wur.nl (J.P.); jurgen.kohl@wur.nl (J.K.); 4 E-nema GmbH, Klausdorfer Str. 28–36, 24223 Schwentinental, Germany; Ehlers@e-nema.de; 5 Strateco OG, Ruckerlberggasse 13, 8010 Graz, Austria; rene.kollmann@strat.eco; 6 Sekem Energy GmbH, Steinberg 132, 8151 Hitzendorf, Austria

**Keywords:** oilseed rape, canola, microbiome, bacterial community, biocontrol, endophytes, *Serratia plymuthica*

## Abstract

Microbiome management is a promising way to suppress verticillium wilt, a severe disease in *Brassica* caused by *Verticillium longisporum*. In order to improve current biocontrol strategies, we compared bacterial *Verticillium* antagonists in different assays using a hierarchical selection and evaluation scheme, and we integrated outcomes of our previous studies. The result was strongly dependent on the assessment method chosen (in vitro, in vivo, in situ), on the growth conditions of the plants and their genotype. The most promising biocontrol candidate identified was a *Brassica* endophyte *Serratia plymuthica* F20. Positive results were confirmed in field trials and by microscopically visualizing the three-way interaction. Applying antagonists in seed treatment contributes to an exceptionally low ecological footprint, supporting efficient economic and ecological solutions to controlling verticillium wilt. Indigenous microbiome, especially soil and seed microbiome, has been identified as key to understanding disease outbreaks and suppression. We suggest that verticillium wilt is a microbiome-driven disease caused by a reduction in microbial diversity within seeds and in the soil surrounding them. We strongly recommend integrating microbiome data in the development of new biocontrol and breeding strategies and combining both strategies with the aim of designing healthy microbiomes, thus making plants more resilient toward soil-borne pathogens.

## 1. Introduction 

*Verticillium longisporum* (C. Stark) Karapapa, Bainbridge and Heale is an important soil-borne pathogen causing wilting diseases in a broad range of plant species of the *Brassicaceae* family [1]. Since it was first detected in Germany in 1960 [2], the fungus has been spreading around the globe, and is now reported as a high-risk pathogen worldwide: e.g., in Europe [1,3,4,5], Russia [6], and Canada [7]. *V. longisporum* is a close relative of *Verticillium dahlia,* its host range is, however, restricted to crucifers and it possesses unusually long conidia. This unique morphology earned the pathogen its first name: *V. dahliae* var. *longisporum* [1]. This fungus has an exceptional evolutionary history: it is a diploid hybrid representing three different species. These species evolved on three different occasions by hybridization between four ancestral lineages, one of which had an endophytic lifestyle [8]. Interestingly, the virulence towards the plants of their respective hosts varies strongly between the *Verticillium* strains, ranging from being strongly virulent to avirulent [9,10,11]. In the avirulent cases, *Verticillium* can often still be found in a given plant’s tissues and thus act rather more like an endophyte than as a pathogen [12]. The endophytic lifestyle of some *Verticillium* spp. have been documented in diverse plant species since the 1960s [13,14,15]. Avirulent endophytical strains of *Verticillium* were even used as biological control agents against *Ophiostoma ulmi* and *V. dahliae* [16,17,18]. Moreover, in recent microbiome studies analyzing healthy, asymptomatic plants, *Verticilllium* is one of the most ubiquitous genera found in plant’s endosphere [19]. The ability of *V. longisporum* to colonize plants as an endophyte as well as high variation in yield losses due to this pathogen [20,21] raises the question of why its behavior is so variable. 

*Brassica napus* L. (oilseed rape, canola) is an important oil-producing crop with an annual worldwide production of over 75 megatons in 2018 [22]. Rapeseed oil is used for many purposes, such as in human consumption, and as a renewable resource for biofuel and animal feed [23]. While demand increased, production became more intensive. Intensification of agricultural production in recent decades has led to a reduction of crop diversity in oilseed rape producing farming: many farmers now use inputs of fertilizers and pesticides to compensate for the lack of rotation [24,25]. Such an intensive farming practice alters the microbial composition of the soil leading to the reduction of its disease suppressive potential, an overall decrease in microbial diversity and an overpopulation of (potential) pathogens [26,27,28]. It has, for example, been shown that the abundance of some pathogenic fungi in continuously cropped oilseed rape rhizosphere communities was enriched in comparison to those communities in which crop rotations took place [25]. Intensive farming practices that occur in the absence of crop rotation may be the main trigger responsible for *Verticillium* switching from an endophytic lifestyle to a pathogenic one, resulting in a premature senescence and ripening which may severely reduce yields [29]. If that is indeed the case, aside from reconsidering farming practices [25,30], and genetic manipulations of the host [31,32], manipulation of microbiome of the crop and the soil may be a powerful and environmentally sustainable alternative method of suppressing verticillium wilt in oilseed rape and other crops. Microbiomes can be managed either directly by applying: i) microbiome transplants; ii) microbes with plant beneficial properties; iii) microbiota-active metabolites, or indirectly by changing environmental conditions in a way that microbiomes also shift their structure and function from dysbiosis into a healthy state [33]. The manipulation of the seed microbiome, and consequently the microbiome of the plant through seed treatments with beneficial bacteria (biocontrol) can be seen as a promising environmentally friendly solution for combating soil-borne diseases. Microbial seed treatments have been developed to resist a number of crops’ diseases [34], and the global market for biological seed treatment is projected to reach US$ 1.8 billion by 2025 [35]. The biocontrol solutions for soil-borne diseases in oilseed rape, such as verticillium wilt, have been in development for over 20 years [36,37,38,39,40,41,42,43,44,45]. A development of a biocontrol strategy against verticillium wilt in oilseed rape is, however, especially challenging due to the presence of glucosinolates and other antimicrobial substances in all tissues of *Brassica* plants. Those substances which contribute to a plant’s defense against microorganisms and pests [46], shape a very specific microbiome with especially tight microbial networks, which are difficult to manipulate with biocontrol agents [45]. One of the first successful attempts to control verticillium wilt in oilseed rape was the seed treatment with a biological control agent *Serratia plymuthica* HRO-C48 [41]. However, inconsistent effects in the field require a better understanding of the complex microbial networks und translation into efficient strategies for microbiome management.

The objective of this study was to improve current strategies and to search for alternative solutions to control verticillium wilt in oilseed rape. Hereby, we focus on the evaluation of biocontrol strains under diverse conditions as well as on the validation of application strategies. We therefore decided to combine outcomes of our previous studies and our established strain collection [42,43,47,48,49] with novel results. This allowed us to showcase the highlights of the successful selection strategy for the environmentally friendly seed treatment with a focus on understanding verticillium wilt as a microbiome disease.

## 2. Results and Discussion

### 2.1. Selection Step One: Soil-Free In Planta Tests for a Preliminary Selection of Suitable Verticillium Antagonists

Over 500 bacterial strains were isolated from the endosphere of *Brassica* plants and tested for their antagonistic activity against *V. longisporum* and for plant growth promotion of oilseed rape seedlings [42]. We selected three promising endophytes, *Serratia plymuthica* F20, *Pseudomonas fluorescens* F2 and *Pseudomonas protegens* F37 for this study. Two further strains, *S. plymuthica* HRO-C48 and *Paenibacillus polymyxa* Sb3-1, were added to the selection as potential biocontrol agents due to their strong antifungal activities in vitro and in vivo [41,43,44,49,50]. All selected *Verticillium* antagonists were screened using an in planta soil-free assay: the bacterial strains were applied to the seeds of oilseed rape, and seedlings were grown under gnotobiotic conditions for two weeks without soil (Table 1).

While treatments of oilseed rape seeds with *S. plymuthica* HRO-C48, *S. plymuthica* F20 and *P. protegens* F37 resulted in a non-significant increase of seedlings’ fresh weight, the strongest and the only statistically significant positive plant growth response amongst tested isolates was observed in those oilseed rape seeds treated with *P. fluorescens* F2. The seed treatment with *P. polymyxa* Sb3-1 on the other hand, resulted in a significant decrease in plant biomass (Table 1). Moreover, the germination rate of the seedlings was negatively affected in a significant way by *P. polymyxa* Sb3-1 and *P. protegens* F37 seed treatments (Table 1). While soil-free in planta tests allow for a fast and easy process of differentiation between putatively plant beneficial microorganisms and those that are potentially deleterious to the host plant, the results of those tests do not always reflect the way that bacteria and plants interact under real conditions [44]. Therefore, such preliminary results always have to be confirmed under natural conditions in soil as discussed below.

### 2.2. Selection Step Two: Soil-Free In Planta Tests for a Selection of Verticillium Antagonistic Consortia

The use of a combination of strains, so-called microbial consortia, is recommended as a promising strategy for development of plant biostimulants and biological control solutions [51,52]. In order to select antagonistic consortia, we studied two opposing examples: *P. fluorescens* F2, which demonstrated a strong plant growth promotion effect in a soil-free system and *P. protegens* F37, which showed an effect on seedling’s germination that was significantly negative (Table 1). Each of these two strains was applied to the seeds of oilseed rape in combination with the most studied and commercially used strain *S. plymuthica* HRO-C48 [41,53,54]. After two weeks of growth in sterile germination pouches, we compared changes in the biomass of seedlings that were treated with a combination of strains with the biomass of seedlings in control treatments, where strains were applied separately. We observed a greater than two-fold reduction in the plant growth promotion effect of the *P. fluorescens* F2 when it was applied in combination with HRO-C48 (Table 1). This reduction in the plant growth promotion effect was statistically significant. The positive effect of *S. plymuthica* HRO-C48 on the seedlings was insignificantly reduced, when this strain was applied in combination with F2. The strong reduction of the efficacy of the F2 when it was used in combination with HRO-C48 indicated that it would not perform well under natural conditions in which a large number of other microorganisms are present. This strain was therefore excluded from further trials. The combined treatment of *P. protegens* F37 with HRO-C48, on the other hand, resulted in a significant two-fold increase of the seedlings’ biomass compared to the singular application of F37 to the seeds. The plant growth promoting effect of HRO-C48 increased non-significantly due to the co-treatment with F37. This result allowed us to conclude that *P. protegens* F37 could be a good “team worker” alongside of other co-inoculants and potentially in combination with indigenous seed and plant microbiota, and thus it was included in the further selection strategy. In conclusion, we determined that the effect of single strain’s inoculation on the growth of plants is strongly dependent on other microorganisms present in the inoculum, whereby the effect of a single strain on the host plant may be enhanced either by increasing or decreasing plant biomass, depending on the co-inoculant. This kind of potentially either/or scenarios, in terms of synergistic versus deleterious effects on the members of antagonistic consortia applied to a variety of plant hosts have been observed on several occasions [55]. Based on the outcomes of this co-inoculation assay we selected following four strains for further investigations: *S. plymuthica* F20, *P. protegens* F37, *S. plymuthica* HRO-C48 and *P. polymyxa* Sb3-1.

### 2.3. Selection Step Three: In Situ Tests in Soil for a Selection of Suitable Verticillium Antagonists

**Figure 1:**
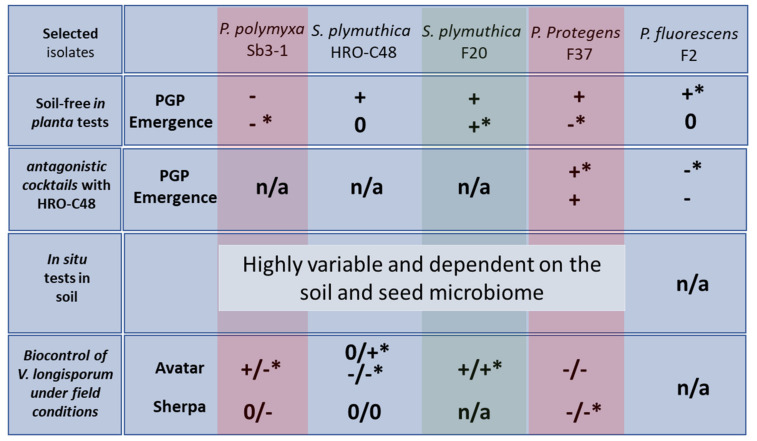
Summary of the selection strategy and results in the development of a novel biocontrol agent against verticillium wilt in oilseed rape. The ”/” sign was used when several fields/conditions were tested. Asterix (*) indicates a significant result under all conditions tested, while n/a means that no data is available. The data derives from this study as well as from the following publications [41,42,43,45].

In previous studies, we were able to show that the effect of biocontrol agents was strongly dependent on the growth conditions of the plants. While *P. polymyxa* Sb3-1 did exhibit a strong negative effect on the oilseed rape seedlings grown in a sterile soil-free system (Table 1), it simultaneously promoted the growth of the plants in sterile soil, and had no significant effect on the biomass of the seedlings grown in non-sterile soil [44]. Such a paradoxical *Paenibacillus*-plant relationship was explained by the shift of the balance between the plant and endophytic bacteria under gnotobiotic soil-free conditions in favor of bacteria, resulting in damage to plant cells [44,50]. In the sterilized soil, on the other hand, this balance may have been shifted towards the plant, probably through production of plant growth hormones and nutrition acquisition by *P. polymyxa* Sb3-1 [50]. A condition specificity of the Sb3-1 seed treatment was further confirmed under disease pressure in pot trials, in greenhouse and in the field [43,45] (Figure 1). We therefore suggest that the choice of the test system for developing a new biocontrol agent is crucial for the outcome of the biocontrol experiments. The balance between bacteria applied to the seed and the composition of the soil microbiome is equally important to the success of the biocontrol study.

A series of in planta experiments under sterile soil-free conditions with singular strains using antagonistic cocktails with HRO-C48 as well as in situ tests in soil and biocontrol experiments against verticillium wilt under field conditions allowed for a selection of a novel potential biocontrol agent *S. plymuthica* F20 (highlighted in green). Two further strains, *P. polymyxa* Sb3-1 and *P. protegens* F37, were considered to be deleterious to oilseed rape (highlighted in red). Due to the varying effects of the selected strains on different plant growth and health parameters, the following simplifications were applied: ”+” sign means that at least one parameter was changed noticeably (more than 10%) in favour of the plants’ health, while other parameters did not change when compared to the untreated control; ”-” sign means that at least one parameter changed noticeably (more than 10%) so that the overall plant health suffered; ”0” means that none of the parameters changed noticeably (more that 10% compared to the untreated control).

### 2.4. Evaluation Step One: Integrating Different Cultivars 

The loss of microbial diversity in soil and consequently in the rhizosphere and endosphere of plants enhances the chances of the emergence of plant pathogens [28,56]. Thus, plants rely on microbial-based defense mechanisms for combating soil-borne diseases [57], which in turn is dependent on the genotype of the plant. The cultivar effect of the biocontrol treatments was observed in various crops such as, for example, potatoes or wheat [58,59,60]. The genotype of the plant was recently shown to be heavily involved in shaping the bacterial composition of oilseed rape [61,62]. The authors demonstrated that the differences in bacterial abundance in plant tissues of various oilseed rape cultivars were predominantly associated with resistance and susceptibility of each cultivar to *V. longisporum* rather than with the health status of the plants (infected vs. non infected) [61,62]. Abuamsha et al. also observed a strong variation in the degree to which different oilseed rape cultivars were affected by the use of biocontrol agents controlling verticillium wilt [63].

The role that the microbiome plays in oilseed rape’s resistance towards *V. longisporum*, was thoroughly investigated in our recent study [45]. Special emphasis was placed on examining the seed microbiome. Seeds contain not only the genetic traits of the mother plant, they also include its essential microbiota [64]. The seed microbiota of oilseed rape was therefore considered to be a crucial factor in determining plant health, strongly influencing (either positively or negatively) the ability of plants to resist soil-borne diseases such as verticillium wilt [45]. The structure of the seed microbiome was consequently regarded as an important factor in the development of colonization resistance against pathogens. The Avatar cultivar of oilseed rape was found to exhibit the lowest bacterial abundance and the smallest degree of microbial diversity. It also had the highest abundance of potential pathogens among the three cultivars studied within the seeds. Simultaneously, the Avatar cultivar responded more strongly than other cultivars to the bacterial inoculation of its seeds. Avatar was the only cultivar to show either no effect or a mildly positive antifungal effect when its seeds were treated with *P. polymyxa* Sb3-1. The effect of the same bacterial treatment on the plants of the other cultivar tested (Sherpa) was rather negative under field conditions [45] (Figure 1). Similar observations were made when seedlings germinated from the treated seeds were grown under gnotobiotic soil-free conditions: Avatar seedlings responded more strongly to the microbial seed treatments than the other cultivars tested. Moreover, applied strains were able to colonize the seeds of the Avatar in significantly higher quantities than the seeds of the other cultivars [45]. 

Based on the evidence illustrated above, we suggest that the breeding of oilseed rape resulted in a diversified microbiome [64], especially within the seeds and rhizosphere. Consequently, variations in the susceptibility of each cultivar towards *V. longisporum* occurred resulting in the variability between the cultivars, to the degree that these cultivars are affected by microbial inocula. The resistance of a microbial community to invasion by either pathogens or beneficials is in correlation with its level of diversity as a result of enhanced competition for resources and available niches [65]. The manipulations of the seed microbiome through biocontrol agents are therefore strongly dependent on the indigenous microbiome of the seed, with the diversity of the seed microbiota being the most valuable criterion [66].

**Figure 2:**
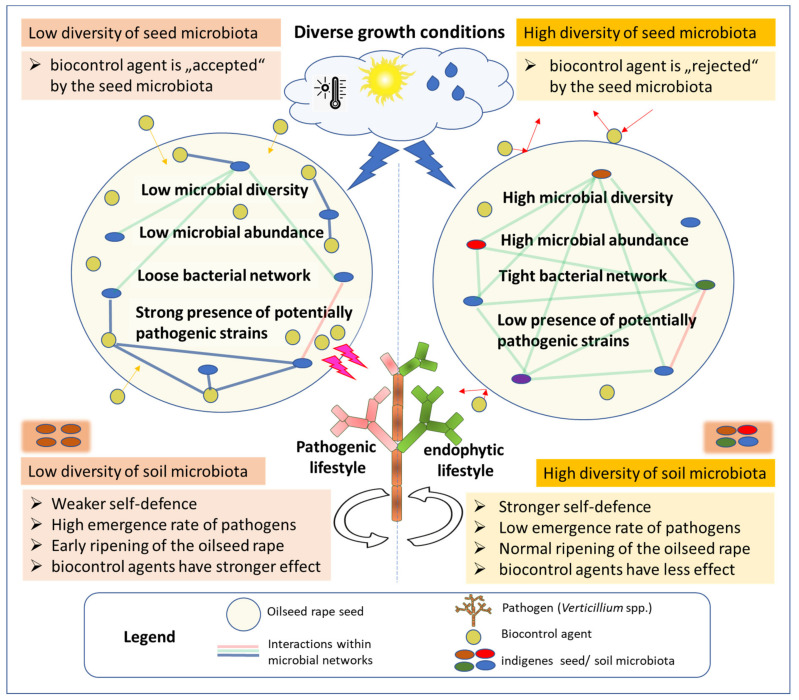
Microbiome interactions and disease outbreaks and suppression.

Thus, based on our data and an extensive literature research, we suggest the following postulates concerning the ways in which the composition of indigenous seed microbiota influences cultivar specificity of the biocontrol agents: (1) low bacterial abundance and reduced diversity within the seed, as well as the loose character of microbial networks and the presence of potentially pathogenic microbial strains increase the possibility of the successful manipulation of the seed microbiota with a suitable biocontrol strain; (2) cultivars whose seeds are characterized by their having high bacterial abundance and diversity, tight bacterial network, and low amount of potential pathogens would potentially have a rather strong self-defense against pathogens. Such cultivars would therefore also be less positively affected by biocontrol agents. These postulates are schematically illustrated in Figure 2.

The schematic shows how the microbial composition of the oilseed rape seed and interactions within microbial networks (indicated as red and green lines) may influence how well the biocontrol strain can be integrated into the microbial network of the seed (new connections are shown as blue lines). This, in turn, affects the efficacy of the seed treatment against soil-borne fungal pathogens, such as *V. longisporum*. Following this schema, a biocontrol agent is more likely to be incorporated into the seed microbiota, when the indigenous seed microbiota has one or several of the following qualities: low microbial diversity, low microbial abundance, loose bacterial network and strong presence of potentially pathogenic strains. On the other hand, high microbial diversity and abundancy, a tight microbial network, and a low presence of potentially pathogenic strains within the seed are signs that a cultivar has a stronger self-defense against pathogens. In such a situation a newly applied biocontrol agent is more likely to be “rejected” by the indigenous seed microbiota and thus have little to no effect on the plant’s health. The various growth conditions of the plants affect these mechanisms and therefore strongly influence the outcome of the biocontrol action.

### 2.5. Evaluation Step Two: Integrating Seed Microbiome Studies 

Domestication and intensive agricultural management resulted in the loss of diversity within the seed microbiota, which has consequences for *One* Health-related issues [19,64]. Thus, it was suggested that bacterial seed treatments may help to restore microbial diversity [19], while compatibility between microbial inoculants and indigenous seed microbiota on a functional level was suggested as an important criterium for the selection of potential biocontrol agents [66]. In our recent study we found that Avatar seedlings responded more strongly to the treatments with *Pseudomonas* spp. in comparison to other bacterial treatments. The colonization of the Avatar seeds by *Pseudomonas* spp. was also significantly stronger than colonization by other tested microorganisms [45]. Moreover, these bacterial species were found in a significantly lower abundance in Avatar seeds compared to the bacterial abundance in the Sherpa seeds. On the other hand, the higher occurrence of *Burkholderia* spp. in both oilseed rape cultivars was suggested to be responsible for the absence of its effect on the seedlings’ growth [45]. 

Following this approach, we speculate that adding the beneficial strains that are missing in the seed microbiota to the seeds of the domesticized agricultural plants allows newcomers to occupy free niches in the plant, thus increasing the overall diversity of the seed microbiota and making the plants more resilient towards invasions by pathogens (Figure 2). Therefore, compatibility of the chosen biocontrol strain with the composition of seed microbiome may be crucial for the outcome of the experiment.

### 2.6. Evaluation Step Three: Analyzing Biocontrol of Verticillium Wilt under Field Conditions

The inconsistency of efficacy of various biocontrol agents when introduced under commercial field conditions has been reported on many occasions [67,68,69,70]. In our previous field and greenhouse trials we also observed a strong variability in biocontrol efficacy [45] (Figure 1). In these trials a number of biocontrol agents were applied to the seeds of up to three oilseed rape cultivars (Avatar, Sherpa and Traviata) and plants were grown under diverse climatic conditions in various field locations. In an effort to compliment previously collected data, we conducted a series of field experiments with the selected strains *P. protegens* F37, *S. plymuthica* HRO-C48 and *S. plymuthica* F20. Each of these strains, either on its own or in a combination with HRO-C48, exhibited a positive effect on the growth of oilseed rape seedlings under sterile soil-free conditions. The selected strains showed a variable, yet overall positive effect on the seedlings in two greenhouse trials (data not shown). The ability of these strains to suppress verticillium wilt was evaluated in two consecutive field trials in Sweden, with two separate field locations each. For the winter trial, the seeds of Avatar and Sherpa oilseed rape cultivars were treated with *P. protegens* F37. Avatar was previously shown to be the most susceptible to the biocontrol treatments, of all cultivars tested. It was therefore additionally assessed in a summer field trial with the two most promising *S. plymuthica* strains, F20 and HRO-C48, applied separately to the seeds. In the winter field trial, the treatment of seeds of the Sherpa cultivar with *P. protegens* F37 resulted in a significant reduction in plant numbers and in the total yield compared to the untreated control in one of the fields (Lockarp) (Table 2). 

The same treatment of the Avatar cultivar had very variable effects on the plants’ growth and health, however none of them was significant. No significant health differences between the treated plants and the untreated control plants of both cultivars grown in the Stångby location could also be observed. The significant reduction of the seedling’s emergence rate in the *S. plymuthica* HRO-C48 treated plants of Avatar cultivar in one of the fields (Kärrarp) was an unexpected finding. The *V. longisporum* infection rate of those plants was, however, mildly reduced, in comparison to the untreated control. On the other hand, *S. plymuthica* F20 showed a stable reduction of verticillium wilt symptoms in the oilseed rape Avatar cultivar in both field locations, one of which was significant (Table 2). This strain was therefore chosen as it showed greatest potential as a novel biocontrol agent for oilseed rape against verticillium wilt. Further studies of the *S. plymuthica* F20 interaction with *V. longisporum* in various oilseed rape cultivars under diverse environmental conditions are necessary to confirm the consistency of its antifungal effect in planta.

The variability of efficacy of biocontrol agents is generally attributed to climatic variations (temperature, humidity, radiation) encountered in field conditions, a lack of ecological competency of the biocontrol agent, the unstable quality of the formulated product and to specific traits of the chosen antagonistic microorganism [69]. In addition to the factors mentioned above, we speculate that variations in the composition of soil microbiota and specifics of the bacterial composition of the seeds strongly influence the efficacy of biocontrol agents. The more diverse the microbiome of the soil, the greater its own biocontrol efficacy [28,71], and the lesser the effectiveness of the additional biocontrol agents. The composition of the indigenous seed microbiota plays a significant role in biocontrol by “choosing” either to accept or reject the strain applied to the seed, and thereby influences the outcome of the biocontrol experiment (Figure 2). In summary, the effect of biocontrol treatments varies depending on the chosen field site and cultivar: indigenous seed and soil microbiota may play a crucial role in the way biocontrol agents affect host plants.

### 2.7. Evaluation Step Four: Visualization of the Interaction between the host plant, Verticillium and its Bacterial Counterpart using Confocal Laser Scanning Microscopy (CLSM) 

**Figure 3:**
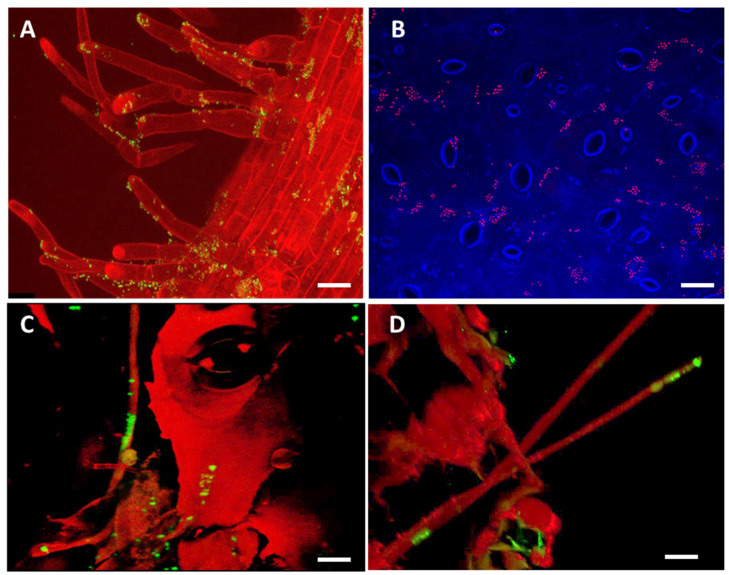
Visualization of a three-way interaction between the host plant, antagonistic bacterium and a pathogenic fungus. The CLSM images show the 28-day old oilseed rape seedlings whose seeds were bioprimed with *gfp*-labelled *S. plymuthica* HRO-C48—visible as green or red dots (**A**,**B**). For the images (**C**,**D**), the seedlings were additionally infected with *V. longisporum.* In (**C**,**D**), the BacLight LIVE/DEAD stain was used to visualize alive (green) and dead (red) bacterial cells. Bar represents 25 μm.

We published stunning images of the rape seed´s microbiome and of biocontrol agents colonizing seeds and various tissues of oilseed rape in our previous studies [44,45]. In order to gain deeper insight into how our selected biocontrol agents interact with *V. longisporum* and with the host plant, we visualized a three-way interaction using CLSM. For this test *S. plymuthica* HRO-C48 was chosen as a representative of a successful biocontrol agent against *V. longisporum* in oilseed rape. Cells of *gfp*-labelled *S. plymuthica* HRO-C48 were applied to the seeds of oilseed rape (cultivar Traviata) and the seedlings were grown for 28 days under gnotobiotic soil-free conditions. In order to study the three-way interaction, the seedlings were additionally infested with *V. longisporum* ELV25, while the control plants were left without the pathogen. We found that HRO-C48 colonized both the phyllosphere and the rhizosphere of the oilseed rape in large amounts in the absence of *V. longisporum*. Bacterial cells were found in large clusters surrounding plant cells (Figure 3A,B). In the *Verticillium*-infested plants, on the other hand, we found large numbers of bacterial cells directly associated with the fungal hyphae, in addition to bacterial clusters associated with the plant tissues (Figure 3C,D). When used in the interpretation of bacterial-fungal interaction the CLSM technique does not allow the viewer to clearly distinguish between synergistic or antagonistic interactions. The ability of HRO-C48 to produce antifungal substances such as pyrrolnitrin or chitinases [72,73] allows us to speculate that the images we observe in Figure 3B,D illustrate how the potential biocontrol agent *S. plymuthica* HRO-C48 may inhibit the growth of fungal pathogens.

The close proximity of the biocontrol agent to the target microorganism we observed in the images may allow the antagonistic substances produced by the bacterium to reach the cells of the fungus in the most efficient way, directly through the cell wall. There are studies available in which an antagonistic interaction between a bacterium and a pathogenic fungus was visualized using CLSM on a agar plate [74]. We believe that our attempt to visualize the three-way interaction ad planta directly on plants of agricultural importance, is the first of its kind.

### 2.8. Assessment: Analyzing the Ecological Footprint 

The application of beneficial microorganisms to seeds is known to be an efficient mechanism for the placement of microbial inocula into soil where they will be well positioned to colonize seedling roots and protect against soil-borne diseases and pests [34]. The comparison of seed inoculation techniques and inoculant concentrations was thoroughly evaluated in previous studies. When comparing seed applications via pelleting, film coating and bio-priming in various inoculant concentrations, none of the variables showed significant influence on the abundances of *S. plymuthica* HRO-C48 in the rhizosphere after 30 days of plants’ growth in a greenhouse [41]. This observation was confirmed by our recent study in the field. There were no significant differences in the germination of oilseed rape seedlings and *V. longisporum* infection rates between the treatments where HRO-C48 was applied using bio-priming compared to the seed coating method [45]. The 100-fold variation of the *P. polymyxa* Sb3-1 concentration applied to the seeds also did not result in any significant alterations either in the seed or root colonization rates or in the seedlings’ growth parameters, when oilseed rape seedlings were grown in soil with and without disease pressure [43,44]. One of the reasons for the lack of significant alterations may be that *V. longisporum* infection affects the crop in the later stages of plants’ growth. By that time, biocontrol agents have already had sufficient time to colonize the plants’ tissues independent of the initial concentration of the inoculum. These observations could potentially contribute to keeping the concentration of inoculum for the commercial processes low and to making the best possible decisions about the choice of cost and energy efficient application types. This will potentially reduce the cost and ecological footprint of bacterial seed treatment for commercial applications.

The use of biofertilizers, and especially biological seed treatments, has been suggested as a reactive measure to fight agriculture’s growing ecological footprint [35]. Application of the beneficial microorganisms directly to the seeds shifts the focus of crop management strategies from the overuse of chemical fertilizers to proactive early intervention, thus reducing the overall environmental burden of chemical fertilizer dependent crop production. Biological seed treatment helps in suppressing and controlling pathogen diseases throughout the plant’s life cycle and beyond: the durability of the biocontrol agents’ effects was demonstrated to be longer than that of chemical alternatives [69]. One of the generally desirable characteristics of a good formulation is maximal reduction of its footprint [75]. We therefore decided to evaluate the impact of the bacterial seed treatments against verticillium wilt in oilseed rape on the environment. The quantity of bacterial cells of all strains used in field trials applied to the seeds remained constant. The production method of the bacterial biomass for seed treatments also remained constant for all strains used in the field studies. Biocontrol agent *S. plymuthica* HRO-C48 was chosen as a representative microorganism for the evaluation of the ecological footprint of the bacterial seed treatments against verticillium wilt in oilseed rape because the industrial commercial production of this strain has been very well established for decades. 

**Figure 4:**
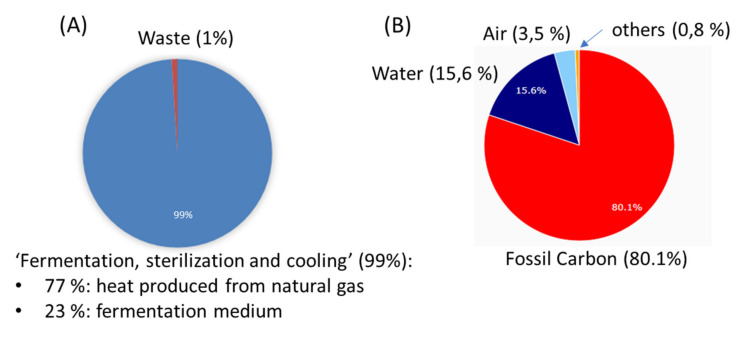
Evaluation of application technologies in terms of ecological footprint. Percentage share of the single process steps (**A**) and the different SPI-categories (**B**) to the overall footprint for *S. plymuthica* HRO-C48 production.

Figure 4A shows the percentage share of the single process steps relevant for the overall ecological footprint in the treatment of oilseed rape seeds with HRO-C48. 

The step ‘fermentation, sterilization and cooling’ was responsible for the overall footprint (99%), while ‘waste treatment’ with only 1% was almost negligible for the total footprint [76]. The heat produced from natural gas was the main ecological hot-spot with almost 77% of the ecological footprint allocated to the ‘fermentation, sterilization and cooling’ step. The residual 23% of the ecological footprint resulted from the fermentation medium itself. The differentiation regarding the Sustainable Process Index (SPI^®^) categories is shown in the Figure 4B. The figure demonstrates that fossil carbon was responsible for the major part (80.1%) of the entire ecological footprint. Of the total footprint area 15.6% were caused by emissions to water and 3.5% by emissions to air. The emissions to soil and the used area were minimal. The overall ecological footprint of the production of HRO-C48 added up to a value of 1.4 m^2^ for the application per ha. This very small ecological footprint resulted from the production of an enormous quantity of cells in the fermentation process (2 × 10^17^ cells per 10,000 l fermenter) compared to the relatively low application rate (5 × 10^11^ cells per ha oilseed rape). In ecological terms the treatment of seeds with bacteria offers another considerable advantage over other microbial application methods. The method of bacterial seed application is carried out with the sowing machine once a year within the sowing process, eliminating the need for additional tractor hours. The application of pesticides in the fields, in particular by the use of machines, is a major detrimental contributor to the cumulative ecological impact (Kollmann, personal communication). In summary, we found that the production of *S. plymuthica* HRO-C48 for the seed treatment against verticillium wilt in oilseed rape is a remarkably environmentally friendly process. Its benefits are derived from the comparatively low application rates of the biocontrol agent in combination with a high number of cells produced during a fermentation process, and from reduction in the need for extra machinery usage. 

### 2.9. Assessment: Analyzing the Ecological Background

The ability of *Verticillium* to act either as a pathogen or an endophyte makes it a unique and challenging target for treatments. This unique ability also raises a question of what exactly the natural function of *Verticillium* as an endophyte is. There is no clear answer to this question exist to date. Based on the ability of *V. longisporum* to induce premature ripening of the oilseed rape [29], we speculate that *Verticillium* spp. is involved in the ripening process of oilseed rape as an endophyte when the microbiome of the plants is well balanced,. A loss of microbial diversity as observed to be the result of intensive agricultural farming of oilseed rape may disturb this microbial balance. As a consequence, a shift of the *V. longisporum* lifestyle from an endophytic to a pathogenic one may occur (Figure 2). Following this shift, *V. longisporum* invades host plants as a pathogen, resulting in a premature ripening of rapeseeds, associated with severe yield losses.

Because the microbial composition of plants is crucial for verticillium wilt outbreaks, and for efficient suppression of the disease, soil and plant microbial diversity are suggested as the keys to suppressing Verticillium outbreaks under field conditions. It was shown that the microbiome plays a crucial role in the suppression of verticillium wilt in other pathosystems [77]. Thus, we recommend re-thinking breeding and biocontrol strategies by integrating microbiome data in the development of new cultivars and biocontrol agents. We suggest that parameters, such as bacterial abundance and diversity within the seed, as well as the character of microbial networks and the presence of potentially pathogenic strains should be taken into account in the process of developing of new cultivars. The greater the diversity of the indigenous bacterial microbiome of the seed, the stronger its resistance to pathogens, and the lesser the need for further interventions will be. Further, we recommend including host seed microbiome analysis in early stages of biocontrol agents’ development. This may help in selecting a suitable biological treatment which is compatible with the indigenous seed microbiome. Such a treatment should enhance overall microbial diversity of the seed by adding the “missing” microbes to the existing microbiota (Figure 2).

## 3. Material and Methods

### 3.1. Bacterial Strains and Growth Conditions 

The following bacterial strains from the strain collection TU Graz, Environmental Biotechnology were used in this study: *P. fluorescens* F2, *S. plymuthica* F20 *and P. protegens* F37, isolated from cauliflower [42] as well as *P. polymyxa* Sb3-1 isolated from agricultural soil [78] and *S. plymuthica* HRO-C48 isolated from the rhizosphere of oilseed rape [41]. The *gfp*-labelled *S. plymuthica* HRO-C48 strain was described elsewhere [44]. The fungal pathogen used was *V. longisporum* ELV25 Stark [1,79] (strain collection TU Graz, Environmental Biotechnology). Bacterial strains were routinely grown on Standard I nutrient agar (NA, SIFIN, Berlin, Germany) at 30 °C.

### 3.2. Co-Inoculation Studies under Sterile Soil-Free Conditions

The effect of the combination of selected bacterial strains on the growth of oilseed rape seedlings was studied using gnotobiotic soil-free assay. The seeds of the winter oilseed rape *Brassica napus* L. partim cultivar Avatar (NPZ) were bio-primed with a combination of *P. fluorescens* F2 and *S. plymuthica* HRO-C48 as well as with a combination of *P. protegens* F37 and *S. plymuthica HRO-C48* following the protocol described in Rybakova et al. [44] with the following modifications. The cell concentration of each strain was separately adjusted with sterile 0.85% (w/v) NaCl to an optical density corresponding to OD_600_ of 10. For the study of the effect of combination of strains on the seedlings’ growth, 5 mL of each of the two preadjusted cell cultures were combined prior to immersion of the seeds in the cell suspension. The bio-primed seedlings were grown in sterile germination pouches (Mega International, Minneapolis, USA) for 14 days as described by Rybakova et al. [44]. The weights of the green parts of the 14-day-old seedlings were compared to the untreated control seedlings and to the weights of the seedlings that were treated with each strain separately. The experiment was carried out in four replicates for each combination of strains with 14 bio-primed seeds from each cultivar. Plant growth promoting effects of the microorganisms and combinations thereof were statistically analyzed using the IBM SPSS program version 20.0 (IBM Corporation, Armonk, NY, USA). The significance of the differences in plants’ weights between the controls treated with one strain each and each combined treatment group was calculated using a pairwise *t*-test with independent samples as described by Rybakova et al. [44]. The decision to make use of the non-parametric Mann-Whitney *U* test as an alternative to the *t*-test was based on assessment of the distributions of variables (normal versus non-normal). Data was expressed as the geometric mean  ±  standard deviation.

### 3.3. Field Trials 

Over the course of the two years, two field trials were conducted with *P. protegens* F37, *S. plymuthica* HRO-C48 and *S. plymuthica* F20. The seeds of oilseed rape *Brassica napus* L. partim cultivars Avatar or Sherpa cultivars (NPZ, Germany) were treated with each bacterial strain using a bio-priming method. It involved treating the seeds with bacterial suspension for 2 h under agitation followed by drying in a fluid bed dryer at 38 °C. Control treatment was suspended in 0.1 M MgSO_4_ × 7H_2_O only. During the first year (winter trial 2016–2017), the seeds of Avatar and Sherpa cultivars were treated with *P. protegens* F37 and the field experiments were carried out at the Stångby and Lockarp sites of oilseed rape production, Sweden (55° 47′ 11.4′’ N13° 9′ 54.7′’ E and 55° 32′ 22.5117′’ N13° 0′ 2.2931′’ E, respectively). Plot size was 3 × 12 m in both trials. The trials had four replicates of each treatment in a randomized block design. In both years the two cultivars as well as the treatments were randomized within four blocks. Both fields had a history of verticillium wilt disease; they are known to be naturally enriched with *V. longisporum*. The number of plants were counted after 50% germination and then again counted after fully emerging. The plants were counted a third time in the spring in order to determine how many plants had died over the course of the winter and yield was determined by harvesting the plots with a parcel combine harvester. Symptoms of verticillium wilt were assessed, and the proportion of infested plants was calculated.

The second field trial (summer 2017) was carried out with the Avatar cultivar of oilseed rape, as this cultivar was more sensitive towards biological treatments in comparison to other tested cultivars in previous studies [45]. The seeds of Avatar cultivar were treated with *S. plymuthica* HRO-C48 and *S. plymuthica* F20 and the field experiments were carried out at the Kärrarp and Selleberga site of oilseed rape production, Sweden (56° 9′ 29.6359′’ N12° 59′ 28.0549′’ E and 56° 4′ 57.4′’ N12° 56′ 12.9′’ E, respectively). The Selleberga field had a history of verticillium wilt disease, while Kärrarp site was artificially inoculated with *V. longisporum* one year before this experiment as described by Rybakova and colleagues [45]. Plot size was 2.5 × 12 m in both trials. The trials had four replicates of each treatment in a randomized block design. The number of plants were counted after 50% germination and then again counted after fully emerging. Symptoms of verticillium wilt (stem striping, chlorosis and wilting of half leaves) were assessed and the proportion of infested plants was calculated. Since these trials were sown with a winter cultivar (Avatar) in the spring, the seeds were not yet fully ripened by the beginning of winter frost and the total yield was therefore not estimated. The significance of the differences in plants’ weights between the controls treated with one strain each and each combined treatment group was calculated using a pairwise *t*-test with independent samples at a significance level of *p* = 0.1, due to a high variation of values observed for field trials. The decision to make use of the non-parametric Mann-Whitney *U* test as an alternative to the *t*-test was based on assessment of the distributions of variables (normal versus non-normal). Data was expressed as the geometric mean  ±  standard deviation.

### 3.4. Visualization of the Bacterial and Fungal Interaction Ad Planta using CLSM

CLSM was used to study in vivo colonization patterns of *S. plymuthica HRO-C48* and *V. longisporum* ELV25 cells in oilseed rape. Seeds of oilseed rape (cultivar Traviata) were bio-primed with HRO-C48 cells labelled with fluorescent marker *gfp* [44], and seedlings grown in germination pouches for 28 days as described above (3.2). This cultivar was chosen as it was routinely used for our gnotobiotic studies in previous studies [44]. The roots of 1-week old seedlings were inoculated with *V. longisporum* ELV25 using a root dipping method as described by Rybakova et al. [43]. In short, the roots of the plants from each plastic container were cut and dipped for 30 min in 50 mL of the solution containing 5 × 10^6^ *V. longisporum* blastospores per mL. The negative control plants were treated the same way but immersed into a sterile water instead of the blastospore solution. After the root dipping, the plants were grown for further three weeks under the same conditions as before. After the end of the incubation period the roots of the plants were examined using CLSM to study the bacterial colonization and the interaction with the pathogenic fungus ad planta. Thin slices of the base, the root tip and the leaves of the plants were investigated. For the detection of GFP-labelled *Serratia* cells (negative control plants as well as for the control tests on the infested plants), an excitation at 488 nm laser line wavelength and a detection at a maximum of 635 nm was used. The plant tissues (autofluorescence) were monitored using a 405 nm laser line wavelength and detected at 425–490 nm. For the visualization of bacterial-fungal interaction ad planta the LIVE/DEAD^®^ BacLightTM Bacterial Viability Kit (Thermo Fisher Scientific, Waltham, MA, USA) was used following the manufacturer’s protocol. The living bacteria and fungi were monitored with the same excitation and emission as was used for GFP (405/425–490 nm for excitation/detection, respectively). To make the dead cells visible a 405 nm laser line wavelength and a detection at a maximum of 532 nm was used. The plant tissues (autofluorescence) were observed using a 405 nm laser line wavelength and detected at 425−490 nm. Confocal stacks were acquired with Z-step of 0.4–0.5 μm and sequential activation of laser lines/detection windows. Maximum projections of an appropriate number of optical slices were applied to visualize the root sections (confocal stacks).

### 3.5. Assessment of Ecological Footprint of the Treatment

The ecological footprint of the production of the seed treatment with *S. plymuthica* HRO-C48 as described by Abuamsha and colleagues [80] was assessed using the Life Cycle Assessment method. Life Cycle Assessment has become an integral part of planning processes in industry as well as for energy and infrastructure systems [81]. An essential part of any Life Cycle Assessment which evaluates the pressure on the environment executed by the life cycle of a product or service is the Life Cycle Impact Assessment [82]. The SPI is a Life Cycle Impact Assessment method evaluating environmental impacts generated by individual processes steps in a life cycle. This method is a member of the ecological footprint family and is compatible with the procedure of the life cycle analyses described in the EN ISO 14040 series [83]. The SPI calculates the ecological footprint as the cumulative area to embed the whole life cycle of an industrial process sustainably into the biosphere. It describes relevant ecological pressures of a process including all emerging emissions, the pre-chain and product usage [84]. Material and energy flows of a product or service, which are extracted from and dissipated to the ecosphere, are compared to natural flows [85]. The SPI method has been implemented in the open access tool SPIonWeb, freely available on http://spionweb.tugraz.at/. With this tool it is possible to create whole life cycles by building up process chains, which can be updated and improved easily. As a result, the user gets the SPI Footprint, CO_2_-life-cycle-emissions and the global warming potential of the whole life cycle.

## 4. Conclusions

The effect of biological inoculants on plant health strongly depends on the cultivar and microbiome composition indicating the coordinated interplay within the plant holobiont. Moreover, the effect is strongly influenced by the chosen method (in vitro, in vivo, in situ); all factors have to be considered in evaluation strategies. Holobiont interactions are also crucial as *Verticillium* act either as a pathogen or as an endophyte; due to the loss of microbial diversity as a result of intensive agricultural farming of oilseed rape, the plant-microbiome balance is disturbed. As plants’ microbiota composition is crucial for verticillium wilt outbreaks as well as for the efficient suppression of the disease, soil and plant microbial diversity are suggested as keys to suppress Verticillium outbreaks under field conditions. In spite of the variability of the effects and difficulties in managing *Brasssica*’s microbiome, we were able to develop a novel promising biocontrol agent, *Brassica* endophyte *Serratia plymuthica* F20. By targeting the seed, the new-coming beneficial strains get a chance to occupy the specific niches in the seed even before it gets in contact with the diverse competitive microbiota of the environment. This kind of a treatment in the case of verticillium wilt of oilseed rape revealed itself to be especially environmentally friendly.

## Figures and Tables

**Table 1:** Effect of the preselected bacterial strains and combinations thereof on the two weeks old oilseed rape seedlings grown under sterile soil-free conditions.

Parameter	Singular Bacterial Treatment					Dual Treatment	
Treatment/Parameter	*S. plymuthica* HRO-C48	*P. polymyxa* Sb3-1	*S. plymuthica* F20	*P. protegens* F37	*P. fluorescens* F2	F37 + HRO-C48	F2 + HRO-C48
Fresh weight of 10 plants [mg] ^(1)^	464 ± 38 (409 ± 9.1)	260 ± 30 * (409 ± 9.1)	509 ± 86 (337 ± 72)	370 ± 26 (337 ± 72)	758 ± 187 ^*^(337 ± 72)	627 ± 45(591 ± 34)	356 ± 58 (591 ± 34)
Germination (%)	96 (100)	75 ^*^ (100)	86 (75)	66 ^*^ (75)	73 (75)	95 (93)	82 (93)
Reference	[44]	[44]	[42]	[42]	[42]	This study	This study

^(1)^ Effect of the bacterial treatments under sterile soil-free condition is expressed as the average fresh weight of the green parts of 10 two-weeks old seedlings. As presented data derives from different experiments, the values for the untreated control are given in brackets after each treatment value. The asterisk (*) denotes values that were significantly different from the non-treated control group values (*p* < 0.05) defined using pairwise *t*-test or non-parametric Mann–Whitney U-test, depending on the distribution of the samples.

**Table 2:** Effect of the bacterial strains *P. protegens* F37, *S. plymuthica* F20 and HRO-C48 on the fitness and health of oilseed rape plants (Avatar cultivar) under field condition.

Field Trial 2016–2017 (Winter)	Number of Plants per m^2^		% Verticillium-Infected Plants		Yield (kg/ha)	
Strain/oilseed rape cultivar	Lockarp	Stångby	Lockarp	Stångby	Lockarp	Stångby
Untreated/ Avatar	29.3 ± 2.9	37.3 ± 5.2	42.4 ± 4.1	50.3 ± 4.5	4173.7 ± 162.6	4663.2 ± 108.8
F37/Avatar	31.8 ± 2.6	30.8 ± 0.9	48.7 ± 5.3	40.8 ± 8.8	4461.3 ± 101.1	4637.0 ± 114.3
Untreated/ Sherpa	34.3 ± 2.8	34.3 ± 3.6	40.6 ± 3.2	46.0 ± 4.6	4090.2 ± 118.6	4700.4 ± 93.9
F37/Sherpa	8.8 ± 2.1 *	33.5 ± 4.9	34.0 ± 3.2	53.2 ± 11.2	2529.7 ± 164.1 *	4412.2 ± 276.0
**Field trial 2017 (summer**)	**Number of plants per m^2^**		**% Verticillium-infected plants**		**Yield (kg/ha**)	
	Sellersberga	Kärrarp	Sellersberga	Kärrarp		
Untreated/ Avatar	63.5 ± 4.6	29.8 ± 3.2	25.0 ± 2.9	17.5 ± 4.8	n/a	
F20/Avatar	51.8 ± 9.7	22.8 ± 1.4	12.5 ± 4.8 *	10.0 ± 4.1	n/a	
HRO-C48/Avatar	52.8 ± 6.2	18.3 ± 3.4*	20.0 ± 4.1	15.0 ± 2.9	n/a	

The asterisk (*) denotes values that were significantly different from the non-treated control group values of the same field/cultivar (*p* < 0.1) defined using pairwise *t*-test or non-parametric Mann–Whitney U-test, depending on the distribution of the samples.
